# Engineering of ω-transaminases for advanced applications

**DOI:** 10.1007/s00253-026-13888-w

**Published:** 2026-06-04

**Authors:** Uwe Wegner, Nicolaus von Wirén, Gotthard Kunze

**Affiliations:** Department of Physiology and Cell Biology, Leibniz Institute of Plant Genetics and Crop Plant Research (IPK), Corrensstr. 3, 06466 Seeland, Gatersleben, OT Germany

**Keywords:** ω-Transaminases, Rational design, Protein engineering, AI-assisted design, Multi-site mutation, Enzyme cascades

## Abstract

**Abstract:**

ω-Transaminases (ω-TAs) are well-established biocatalysts for the asymmetric synthesis of chiral amines, yet their broader application remains limited by challenges related to activity, substrate scope, equilibrium constraints in asymmetric synthesis, and process compatibility e.g. solvent tolerance. In recent years, research has shifted from enzyme discovery toward targeted development strategies aimed at addressing these limitations. This review summarizes recent advances in ω-transaminase development, with a focus on protein engineering and process-level optimization. Structure-guided and semi-rational mutagenesis approaches continue to provide robust, incremental improvements, while multi-site, activity-driven engineering has proven effective for challenging substrates such as β- and γ-amino acids or bulky amines. Emerging AI- and AlphaFold-assisted workflows increasingly support rational enzyme design by reducing experimental efforts. Beyond enzyme sequence optimization, reaction engineering, multienzyme cascades, and process intensification strategies play a crucial role in improving overall performance and scalability. Genome mining and discovery efforts are briefly discussed as supporting tools for expanding the ω-transaminase toolbox. Overall, studies in the last years show that successful ω-transaminase development relies more and more on integrated approaches combining enzyme engineering with intelligent process design, rather than on isolated optimization strategies.

**Key points:**

•*The state-of-the-art status of ω-TA development is presented.*

•*The shift from enzyme discovery to substrate-based optimization is highlighted.*

•*Modern reaction conditions for more environmentally friendly synthesis are reported.*

## Introduction

ω-Transaminases (ω-TAs) have emerged as key biocatalysts for the asymmetric synthesis of chiral amines, which are essential building blocks in the pharmaceutical, agrochemical, and fine chemical industries (Cui et al. 2025). Their ability to catalyze the stereoselective transfer of an amino group from an amine donor to a prochiral ketone or aldehyde under mild reaction conditions has positioned ω-TAs as attractive alternatives to traditional chemical amination methods (Israr et al. 2025; Fotiadou and Pavlidis 2025).

Over the past two decades, extensive screening efforts and protein engineering campaigns have led to the identification of numerous ω-transaminases with diverse substrate scopes and excellent stereo-preference, usually > 99% ee (Gao et al. 2025a). As a result, ω-TAs are now considered a relatively mature enzyme class within industrial biocatalysis. Nevertheless, several intrinsic limitations continue to restrict their broader application, including limited activity toward bulky or non-native substrates. Sitagliptin is a prominent example for such substrates. Other limitations are unfavorable reaction equilibria in asymmetric synthesis, inhibition by substrates or products, and challenges related to enzyme stability, tolerance to organic solvents (Meng et al. 2020) and cofactor dependency (Vikhrankar et al. 2024).

Recent years have therefore seen a shift from simple enzyme discovery toward more sophisticated development strategies, combining protein engineering, computational tools, and process-level optimization. Rather than focusing solely on identifying new ω-TAs, current research increasingly aims to enhance catalytic performance, expand substrate scope, and integrate ω-transaminases into efficient and scalable reaction systems.

This review provides an overview of recent advances in ω-transaminase development, with an emphasis on the period from approximately 2020 onward. Particular attention is given to protein engineering strategies, including structure-guided mutagenesis, multi-site optimization, and AI-assisted design, as well as approaches at the process level, such as reaction engineering, cascade design, and process intensification. Genome mining and discovery-oriented studies are discussed only briefly, insofar as they contribute to the expansion of the ω-transaminase toolbox.

### Protein engineering strategies for ω-transaminase development

#### Structure-guided and semi-rational active-site engineering

Random mutagenesis (Rodríguez Núñez et al. 2025) is a rarely used technique in contrast to structure-guided protein engineering (Gao et al. 2025b), which remains one of the most widely applied strategies for improving ω-transaminase performance. With the growing number of crystal structures, 69,486 structures in 2010, 247,280 structures in 2025 (RCSB Protein Data Bank 2026) and the recent development of high-quality structure predictions (Fleming et al. 2025), targeted mutations can be introduced to reshape the active site, modulate substrate-binding pockets, or influence access tunnels leading to the catalytic center (Das et al. 2024; Xiang et al. 2025; Patti et al. 2025).

Several studies demonstrate that relatively small numbers of rationally chosen mutations can lead to measurable improvements in activity, the S17P|T38V variant of an ω-TA from *S. thermophilus* had up to 10.5-fold greater activity compared to the wild type (Wegner et al. 2023b), an ω-TA from *Salmonella enterica* could be improved to a 31-fold greater activity (Yi et al. 2025a, b). Substrate acceptance was also improved in some cases, e.g. Sbv333-ATA was modified to accept bulky substrates (Patti et al. 2025). Moreover, structure-guided mutagenesis has been successfully employed to expand the substrate scope of bacterial ω-transaminases toward bulkier ketones or heterocyclic compounds, often by enlarging or reshaping the so-called “large” and “small” binding pockets characteristic of ω-transaminases (Luo et al. 2024; Chen et al. 2025; Cai et al. 2025; Zhu et al. 2025).

In many cases, the observed improvements arise primarily from enhanced substrate accommodation rather than from fundamental changes to the catalytic mechanism. Notably, these approaches tend to yield incremental but robust gains, making them particularly attractive for industrially relevant enzymes where stability and reliability are as important as maximal activity (Duan et al. 2024).

Studies combining homology modeling or AlphaFold-derived structures with docking and limited mutational screening exemplify this trend, illustrating how modern structural tools can streamline semi-rational enzyme optimization without requiring exhaustive directed evolution campaigns (Qiu et al. 2024).

#### Multi-site mutagenesis and activity-driven optimization

Beyond single-point or pocket-focused interventions, several recent works highlight the continued relevance of multi-site mutagenesis strategies aimed primarily at increasing catalytic activity (Zhu et al. 2023; Liu et al. 2024b, 2024a) or stability against harsh reaction conditions (Zhao et al. 2025). In contrast to strictly structure-guided approaches, these studies often prioritize experimental screening and performance metrics over detailed mechanistic interpretation.

A representative example is the engineering of a thermostable ω-transaminase from *Sphaerobacter thermophilus* for the kinetic resolution of β- and γ-amino acids. Through systematic mutation of multiple residues in and around the active site, substantial improvements in reaction rate (up to 10.5 fold greater specific activity than the wild type) were achieved while maintaining high enantioselectivity, > 99% ee (Wegner et al. 2023b). Importantly, combinations of mutations exhibited additive or synergistic effects, underscoring the value of exploring sequence space beyond isolated single-point changes. Another example is provided by Xu et al. (2024), they achieved a 3.7-fold greater specific activity and a 19.9-fold longer half life time at 45 °C.

Such activity-driven optimization approaches are particularly effective for addressing challenging substrates, including non-canonical amino acids and sterically demanding amines, for which naturally occurring ω-transaminases often show only marginal activity (Xu et al. 2024; Pagar et al. 2024). Although these studies may lack the conceptual novelty of AI-driven design, they demonstrate that classical protein engineering remains a powerful and practical tool for ω-transaminase development.

#### AI- and AlphaFold-assisted enzyme design

Recent advances in protein structure prediction and computational modeling have begun to influence ω-transaminase engineering strategies. In particular, the widespread adoption of AlphaFold has significantly lowered the barrier to structure-based design, enabling researchers to rationalize mutational choices even in the absence of experimental crystal structures, according to the RCSB homepage, there are 248,942 experimental structures and 999,251 AlphaFold derived structures available (at Feb. 13th, 2026). This underlines the emerging importance of computational modeling.

Several studies have combined AlphaFold-derived models with molecular docking (Meng et al. 2021; Ramírez-Palacios et al. 2021; Yi et al. 2025a, b) and limited molecular dynamics simulations to guide mutagenesis of ω-transaminases (Yang et al. 2025; Li et al. 2025c). In these works, predicted enzyme–substrate interactions were used to identify residues likely to influence substrate binding, access channel geometry, or cofactor positioning. Subsequent site-directed mutagenesis confirmed that such computationally informed interventions can yield measurable improvements in catalytic activity and substrate scope.

Despite these successes, the impact of AI-assisted design has so far remained incremental rather than transformative. Improvements achieved through AlphaFold-guided engineering are often comparable in magnitude to those obtained by classical semi-rational approaches. Moreover, most studies still rely on experimental screening to validate computational predictions, highlighting that current AI tools primarily serve as decision-support systems rather than autonomous design engines (Li et al. 2025c).

Nevertheless, the integration of structure prediction, docking, and mutational screening represents a promising development (Ao et al. 2023; Menke et al. 2024; Weigmann et al. 2025). As computational methods mature and training datasets expand, AI-assisted workflows may increasingly complement traditional protein engineering strategies, particularly for reducing experimental effort and narrowing down mutational search spaces.

#### Machine learning–driven optimization of transaminases

The integration of Machine Learning (ML) into biocatalysis has emerged as a transformative strategy to bypass the limitations of traditional directed evolution. By leveraging computational models, researchers can navigate the complex protein fitness landscape more efficiently, optimizing enzymes for enhanced activity, stereoselectivity and stability.

#### Data acquisition and feature engineering

A critical bottleneck in ML-based enzyme engineering is the requirement for high-quality training data. Several studies demonstrate that combining rational design with ML can significantly improve model performance. For instance, focused libraries of amine transaminase (ATA) variants, designed through structure-guided mutagenesis, provide a robust dataset for training predictive models (Ao et al. 2023; Yi et al. 2025a, b).

To translate biological information into a machine-readable format, various encoding strategies are employed:

- **Physicochemical descriptors:** Methods are used to describe the electronic and steric properties of substrates and amino acid residues at specific positions to capture the chemical environment of the active site.
- **Digital sequence coding:** Tools like Fast Fourier Transformation (FFT) and AAindex data are utilized to capture both local and long-range interactions within the protein sequence.
- **Thermodynamic features:** Incorporating changes in binding energy (ΔΔG) as a feature allows the model to predict the stability and affinity of the enzyme–substrate complex more accurately.

#### Algorithmic performance and predictive power

Various algorithms have been benchmarked for their ability to predict biocatalytic outcomes. Gradient Boosting Regression Tree (GBRT) models have shown exceptional precision in predicting catalytic activity, achieving correlation coefficients (R2) as high as 0.905. Other ensemble methods, such as Random Forest and LightGBM, have also proven robust in handling the non-linear relationships between protein sequences and functional outputs.

#### Transfer learning and generalization

One of the most promising aspects of ML in this field is Transfer Learning. The work of Weigmann et al. (2025) indicates that a model trained on one enzyme can be adapted to a different, related protein with minimal additional data, as shown with the enzymes 3FCR and 3HMU. This is achieved by using "protein indicators" or shared descriptors, suggesting that ML models can eventually become generalized tools across different enzyme families.

Table 1 summarizes the three presented ML approaches.

**Table 1:** Summary of three exemplary ML approaches

Focus Area	Activity and selectivity (Ao et al. 2023)	Process stability (Yi et al. 2025a, b)	Sequence-function mapping (Weigmann et al. 2025)
Main objective	Improving *k*_cat_/Km and enantioselectivity	Enhancing tolerance to high substrate loads	Predicting mutation effects from small datasets
Key approach	GBRT models and structure-guided libraries	FFT-based sequence encoding and LightGBM	Transfer Learning between ATA scaffolds
Outcome	2000-fold activity increase	High activity at 60 mM substrate	96% reduction in experimental effort

#### Critical assessment of engineering paradigms

The recent literature reveals a distinct trade-off between different engineering philosophies. Traditional structure-guided rational design remains the gold standard for expanding the substrate scope of ω-TAs toward bulky ketones or heterocyclic compounds. By reshaping the "large" and "small" binding pockets, researchers have achieved significant gains in activity, such as the 31-fold improvement in a *Salmonella enterica* enzyme variant. However, these interventions often focus narrowly on substrate accommodation, sometimes at the expense of global protein stability or "evolvability" for other traits.

In contrast, multi-site and data-driven approaches (including Machine Learning and sequence-guided redesign) show a higher success rate in simultaneously optimizing multiple parameters, such as activity and operational stability. For instance, the sequence-guided redesign of a *Bacillus megaterium* ω-TA resulted not only in a 3.7-fold activity increase but also a remarkable 19.9-fold improvement in half-life at 45 °C. This suggests that considering the broader sequence-function landscape—rather than just the immediate active site—is crucial for developing industrially robust biocatalysts.

Despite the transformative potential of AI- and AlphaFold-assisted workflows, their current impact remains largely decision-supportive and incremental. A major bottleneck is the "the limited availability of high-quality training data” problem: while structural predictions are now highly accurate, the lack of diverse experimental datasets—specifically negative data representing failed mutations—limits the ability of machine learning models to autonomously design "super-enzymes" without extensive experimental validation. At present, ML is best viewed as a decision-support tool rather than a fully autonomous design platform. Its usefulness increases when training data are generated in a systematic and iterative manner, ideally combining structure-guided libraries with experimental feedback. Therefore, recent studies integrate these computational tools with classical semi-rational libraries to narrow down the mutational search space effectively.

Table 2 summarizes different enzyme optimization approaches and provides an example for each technique.

**Table 2:** The shift from traditional directed evolution toward AI-assisted semi-rational design reflects a broader trend in ω-TA engineering aimed at reducing experimental overhead. While structure-guided approaches excel at reshaping binding pockets for bulky substrates, they often struggle to predict mutations that enhance distal stability. Conversely, the emerging use of protein language models (PLMs) and AlphaFold allows for the exploration of sequence spaces previously inaccessible due to a lack of experimental templates, though the validation of these “in silico” predictions remains a critical bottleneck in the development of industrial-grade biocatalysts

Strategy	Focus/approach	Representative enzyme (example)	Activity improvement (vs. wild type)	Advantages	Limitations
Rational design (structure-guided)	Targeted mutations based on crystal structures or models	ω-TA from *Salmonella enterica*	31-fold greater activity	High success rate for specific goals; minimal screening effort	Requires precise structural data; overlooks distal residues
Directed evolution	Random mutagenesis (e.g., epPCR) and screening	*Vibrio fluvialis* transaminase	Up to fivefold greater activity, some variants with sixfold greater thermostability and alkaline pH tolerance	No prior structural knowledge needed; finds unpredictable mutations	Massive screening burden; rarely used compared to rational design
Semi-rational/multi-site design	Combination of structural selection and targeted libraries	ω-TA from *Sphaerobacter thermophilus*	10.5-fold greater specific activity	Leverages synergistic effects; robust for industrial stability	Still requires significant screening to validate combinations
AI- and AlphaFold-assisted design	Use of structure prediction and ML to optimize sequences	*Bacillus megaterium* ω-TA (sequence-guided)	3.7-fold greater activity (and 19.9-fold half-life increase)	Enables design without crystal structures; reduces search space	“Black-box” nature; quality depends on data; often incremental gains

#### Beyond enzyme sequence: process-level optimization of ω-transaminase reactions

While protein engineering plays a central role in ω-transaminase development, it has become clear that many practical limitations arise at the process level rather than from the enzyme itself. Consequently, recent studies place growing emphasis on reaction engineering, system integration, and process intensification to unlock the full potential of ω-transaminase catalysis. To transition from laboratory-scale synthesis to commercial production, the development of ω-TA) processes must address rigorous benchmarks for substrate loading, space–time yield (STY), and biocatalyst reusability. From an application perspective, the most relevant question is not only whether a variant performs better under laboratory conditions, but whether it can sustain high productivity under process-relevant substrate concentrations and operational conditions.

#### Reaction engineering and equilibrium control

A fundamental challenge in ω-transaminas﻿e-catalyzed reactions is the unfavorable thermodynamic equilibrium in asymmetric synthesis. Numerous studies have therefore focused on reaction engineering strategies to shift the equilibrium toward product formation, including the choice of amine donors, removal of coproducts, e.g. the removal of pyruvate via pyruvate decarboxylase (Li et al. 2025b), and optimization of solvent systems (Fotiadou and Pavlidis 2025).

Recent application-oriented studies demonstrate that careful tuning of reaction parameters—such as donor concentration, pH, temperature, and cosolvent composition—can substantially improve conversions even when using unmodified enzymes (Vikhrankar et al. 2024). In particular, the use of excess isopropylamine or alternative amine donors, as well as in situ removal of ketone byproducts, remains a widely applied and effective strategy. To overcome the unfavorable equilibrium of ω-TA reactions, recent strategies have shifted toward the use of 'smart' amine donors. For instance, Dunham et al. (2024) demonstrated that using L-lysine as a donor allows for high conversions because the resulting byproduct undergoes spontaneous cyclization, effectively removing it from the reaction system. Isoproylamine is deaminated to acetone, which can easily be removed from the system by low pressure. Furthermore, the application of Deep Eutectic Solvents (DES) as alternative reaction media has been shown to improve the solubility of challenging substrates while maintaining enzymatic activity (Alcántara and de Gonzalo 2025; Jimat and Syed Putra 2026).

**Fig. 1:**
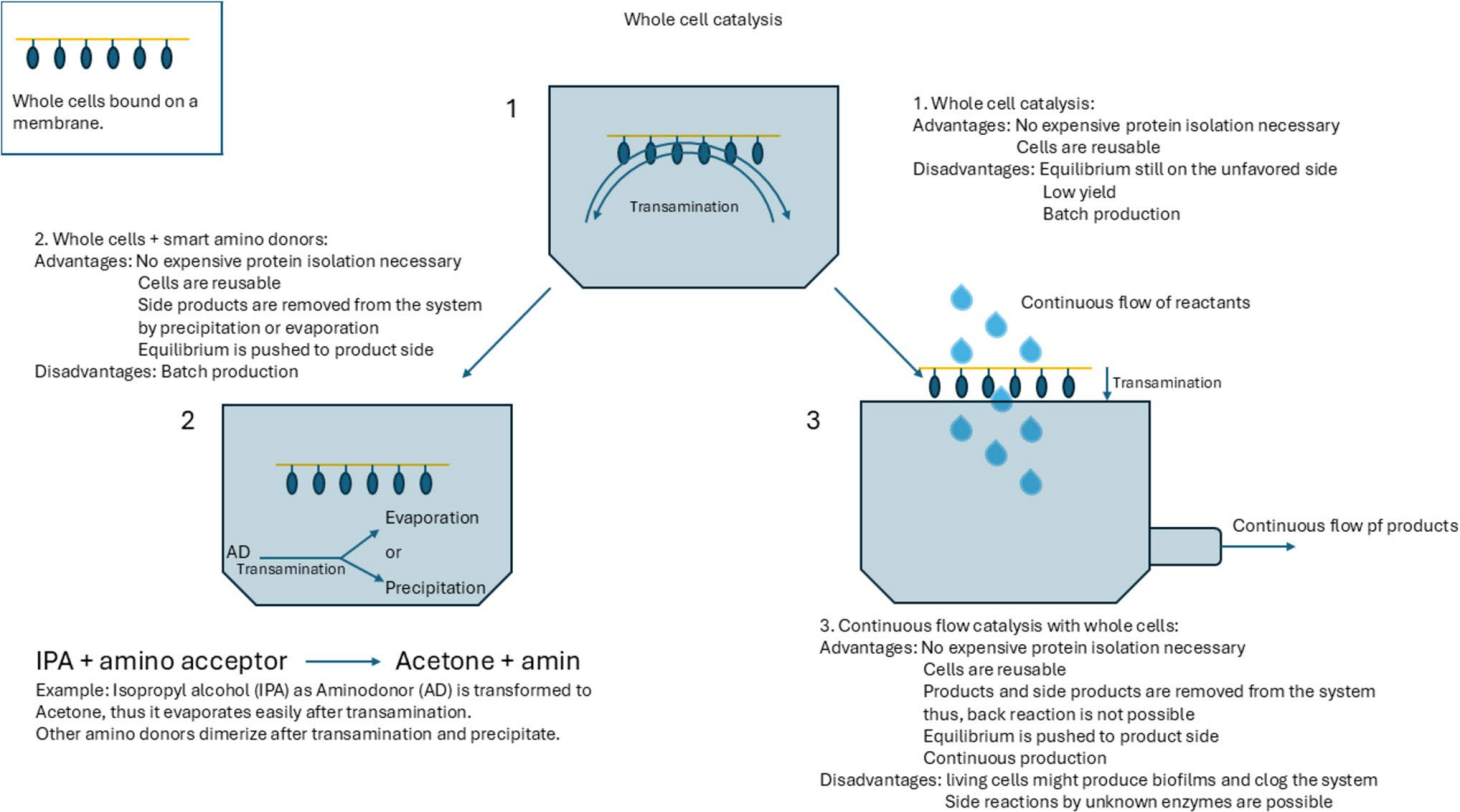
Schematic representation of different methods of industrial transamination. 1, batch production with whole cells in reaction medium; 2, improvement of approach 1 by employing smart amino donors; 3, improvement of approach 1 by switching to a continuous production

Although these procedures are conceptually well established, their continued refinement underscores an important point: enzyme engineering alone is often insufficient to achieve high space–time yields and industrially relevant substrate loadings. Instead, modestly engineered ω-transaminases can frequently be rendered highly effective through appropriate reaction design. Figure 1 summarizes the approaches to push the equilibrium to the product side.

### Multienzyme cascades and system integration

To further address equilibrium constraints and expand synthetic possibilities, ω-transaminases are increasingly embedded within multienzyme cascade reactions. By coupling amination steps to upstream or downstream enzymatic transformations, these cascades enable efficient substrate channeling and continuous regeneration of reaction partners (Wang et al. 2022b).

Recent examples include chemoenzymatic and fully enzymatic cascades for the conversion of renewable feedstocks into value-added amines, such as the transformation of biomass-derived intermediates into functionalized amines (Fornoni et al. 2025; Li et al. 2025a, 2025b). In such systems, ω-transaminases often serve as key stereoselective steps, while auxiliary enzymes drive the overall reaction toward completion.

These studies provide a shift in perspective: ω-transaminases are no longer viewed as isolated catalysts but as modular components within larger synthetic networks. This systems-level integration allows existing enzymes to be applied to challenging transformations without requiring extreme levels of protein engineering.

### Process intensification and continuous concepts

Beyond batch reaction optimization, process intensification strategies and whole cell catalysis (Hagman et al. 2025) are gaining traction in ω-transaminase research. Approaches such as membrane-assisted product removal, continuous-flow operation, and enzyme immobilization (Czarnievicz et al. 2025) aim to improve productivity, stability (Zhou et al. 2025b), and scalability.

A notable example is the integration of ω-transaminase catalysis with polypropylene membrane separation technologies for the asymmetric synthesis of (R)-2-fluoro-α-methylbenzylamine, enabling continuous removal of inhibitory products and improved equilibrium control (Meersseman Arango et al. 2025). Such concepts demonstrate that process engineering can directly compensate for intrinsic enzymatic limitations, offering an alternative or complementary route to extensive protein modification.

Although these intensified systems are still largely confined to laboratory-scale demonstrations, they point toward future industrial implementations in which ω-transaminases operate under continuous or semi-continuous conditions.

### Discovery and toolbox expansion (brief perspective)

Parallel to engineering and process-focused efforts, genome mining and functional screening (Wang et al. 2022a) continue to expand the known repertoire of ω-transaminases (Liu et al. 2025). Recent studies have identified numerous putative ω-transaminases across bacterial genomes and provided baseline characterization of selected candidates (Zhou et al. 2025a).

While these discovery-oriented works contribute valuable sequence diversity (Wegner et al. 2023a; Li et al. 2024; Runthala et al. 2025), their immediate impact on ω-transaminase development remains limited. Most newly identified enzymes exhibit only moderate activity and require subsequent engineering to become practically useful (Wang et al. 2021a). As such, genome mining currently serves primarily as a supporting strategy, supplying raw material for downstream development rather than driving innovation on its own.

Tufvesson et al. (2011) defined several key points for industrial requirements. In Table 3., laboratory scale achievements of ω-TAs are compared with industrial requirements for a cost-effective production. These points were extended by Woodley (2020) by defining, that the biocatalyst productivity should be 20–50 g product per g enzyme to cover the production costs of the enzyme.

**Table 3:** Comparison of laboratory scale key points for ω-TA Performance with industrial requirements

Parameter	Laboratory standard	Industrial requirement	Enabling technology
Substrate loading	10–60 mM	> 200 mM	DES, Smart Amine Donors
Space–time yield	Variable	From 20 g L^−1^ d^−1^ (pharmaceuticals) to 100 g L^−1^ d^−1^ (bulk chemicals)	ML-optimized *k*_cat_, Continuous flow
Conversion	50–90%	> 95%	Equilibrium shifting (e.g., pyruvate removal)
Biocatalyst cost	High (purified)	Low (Whole-cell/Recycled)	Immobilization, whole-cell catalysis
Substrate concentration	Usually mg ml^−1^	> 100 g L^−1^ (fine chemicals)	Immobilization, whole-cell catalysis, continuous flow
Product concentration	Usually mg ml^−1^	> 50 g L^−1^	Immobilization, whole-cell catalysis, continuous flow

These benchmarks demonstrate, that despite all progress in the last decades, ω-TAs or reaction conditions still need further optimization to meet the desired values. Conversion rates are already quite close to the requirements (up to 90% are reached, 95% are desired), but the substrate load is still quite low, here up to 60 mM can be reached, but > 200 mM are needed. A combination of recent achievements, like integrating optimized enzymes as whole cell catalysts into a continuous reaction system, would be a promising approach to meet industrial challenges, but this would still require case by case studies. Next to the mentioned points, there are new challenges to overcome with whole cell catalysts, as living cells tend to form biofilms, which can clog the system, which causes costs for maintenance and interruptions of the production. Also, the reaction speed can be lower in whole cell catalysis, because the reactants have to move through barriers, which do not exist in systems with purified enzymes, this is the so called Mass transfer resistance. The cell membrane is one of the barriers, another hindrance can be the matrix, in which the cells are embedded.

## Conclusions and outlook

The presented advances in ω-transaminase research clearly demonstrate that the field has entered a phase of targeted development rather than broad exploration. While the fundamental catalytic mechanism and general applicability of ω-transaminases are well established, current efforts focus on overcoming persistent limitations related to substrate scope, catalytic efficiency, and process compatibility (Cao et al. 2023; Yi et al. 2025a, b).

Protein engineering remains a central driver of progress. Structure-guided and semi-rational mutagenesis strategies (Wang et al. 2021b) continue to deliver reliable, incremental improvements, while multi-site, activity-driven optimization approaches have proven particularly effective for challenging substrates such as β- and γ-amino acids (Jeon et al. 2022). At the same time, AI- and AlphaFold-assisted workflows are increasingly adopted as enabling tools to guide mutational choices and reduce experimental screening effort, although their impact currently complements rather than replaces classical engineering approaches.

Importantly, recent publications emphasize that many practical bottlenecks are not purely enzymatic in nature. Reaction engineering, cascade design, and process intensification strategies—including equilibrium control, multienzyme systems, and continuous concepts—have emerged as equally important components of ω-transaminase development. These approaches highlight a growing recognition that optimal performance is often achieved through the co-development of enzyme and process.

Looking ahead, future progress in ω-transaminase technology is likely to depend on integrated strategies that combine moderate enzyme engineering with intelligent reaction and process design. Rather than pursuing universal or highly optimized “super-enzymes,” the field appears to be moving toward tailored solutions for specific substrate classes and process requirements. In this context, ω-transaminases are well positioned to remain key biocatalysts for sustainable amine synthesis in both academic and industrial settings.

To summarize the major challenges in ω-TA research; they have a limited substrate scope and insufficient stability under harsh conditions, next to the unfavorable thermodynamic equilibrium. These aspects are already addressed by several studies with great success. Although there is no—and likely will not be—universal enzyme for all purposes, the substrate scope of many enzymes has successfully been widened to increase the number of produceable amines. To make the enzymes even more efficient, the reaction conditions were optimized, to shift the equilibrium to the product side, e.g. by removing the products continuously. Another important point is the limited data set for the training of ML systems. As machine learning is an emerging field, a sufficient database is necessary. For this database, even negative results would be important, to teach the machine, which sequence modifications lead to a loss of activity and/or stability. A better database for ML would lift the ω-TA field to a new level, as AlphaFold did with the availability of structural information. In the past, the focus was on discovering new enzymes. This switched to optimizing sequences for specific interests or to broaden the substrate scope of known enzymes. This was followed by a shift to improve the reaction conditions and reusability. ML has the potential to combine all so far gained information to predict specific enzymes and conditions for advanced applications. For this, more experimental data about the role of all residues is needed, even if an exchange of a specific residue is disadvantageous or has no effect. It is good to publish about successful optimizations, but information about failed or low effect attempts becomes more important. Maybe researchers should be encouraged to publish such data or to make it publicly available, because all ω-TA researchers, no matter if *in-silico* or in the wet lab, would profit from it.

## Data Availability

No datasets were generated or analysed during the current study.
